# Epidemiology and economic burden of measles, mumps, pertussis, and varicella in Germany: a systematic review

**DOI:** 10.1007/s00038-016-0842-8

**Published:** 2016-08-04

**Authors:** Oliver Damm, Julian Witte, Stefanie Wetzka, Christine Prosser, Sebastian Braun, Robert Welte, Wolfgang Greiner

**Affiliations:** 1 Department of Health Economics and Health Care Management, School of Public Health, Bielefeld University, Universitätsstraße 25, 33615 Bielefeld, Germany; 2 GlaxoSmithKline Germany, Prinzregentenplatz 9, 81675 Munich, Germany; 3 Xcenda GmbH, Lange Laube 31, 30159 Hanover, Germany

**Keywords:** Childhood diseases, Epidemiology, Economic burden, Germany

## Abstract

**Objectives:**

Despite the availability of vaccines and the existence of public vaccination recommendations, outbreaks of vaccine-preventable childhood diseases still cause public health debate. The objective of this systematic review was to provide an overview of the current epidemiology and economic burden of measles, mumps, pertussis, and varicella in Germany.

**Methods:**

We systematically reviewed studies published since 2000. The literature search was conducted using PubMed and EMBASE. Also, we used German notification data to give an up-to-date overview of the epidemiology of the four diseases under consideration.

**Results:**

Thirty-six studies were included in our review. Results suggest that there is still considerable morbidity due to childhood diseases in Germany. Studies providing cost estimates are scarce. Comparative analyses of different data sources (notification data vs. claims data) revealed a potential underestimation of incidence estimates when using notification data. Furthermore, several studies showed regional differences in incidence of some of the diseases under consideration.

**Conclusions:**

Our findings underline the need for improved vaccination and communication strategies targeting all susceptible age and risk groups on a national and local level.

**Electronic supplementary material:**

The online version of this article (doi:10.1007/s00038-016-0842-8) contains supplementary material, which is available to authorized users.

## Introduction

Vaccination is regarded as one of the great public health achievements (CDC 1999) and has led to substantial decreases in morbidity and mortality of vaccine-preventable diseases (Roush and Murphy 2007). However, despite the availability of vaccines and the existence of public vaccination recommendations, vaccine-preventable childhood diseases are still a subject of public health debate and research. This is mostly due to outbreaks such as the large measles outbreak in Berlin in 2014/2015 (RKI 2015b).

In Germany, current vaccination recommendations of the Standing Vaccination Committee (STIKO) cover, among others, routine childhood vaccination against measles, mumps, pertussis, and varicella (Table 1). Some of these recommendations have existed for decades and undergone several updates. A detailed description of the history of vaccination recommendations in Germany was published by Klein et al. (2012).

**Table 1:** Measles, mumps, pertussis, and varicella vaccination recommendations and mandatory reporting in Germany (Klein et al. 2012; RKI 2014)

Disease	Introduction of routine childhood vaccination recommendation	Current childhood immunisation schedule	Current adult immunisation schedule	Mandatory reporting since
Measles	FRG: 1974; GDR: 1966 (voluntary), 1970 (mandatory)	11–14 months and 15–23 months (catch-up until 17 years)	One-time vaccination for all adults born after 1970 who are of unclear vaccination status, are unvaccinated, or have received only one vaccination in childhood	2001
Mumps	FRG: 1976; GDR: 1977 (voluntary)	11–14 months and 15–23 months (catch-up until 17 years)	No routine immunisation	2013
Pertussis	FRG: 1969/1991^a^; GDR: 1964 (mandatory)	2–4 months and 11–14 months (catch-up until 4 years); booster 5-6 and 9-17 years	The next due tetanus and diphtheria vaccination as a one-time tetanus, diphtheria and pertussis combination vaccination	2013
Varicella	2004	11–14 months and 15–23 months (catch-up until 17 years)	No routine immunisation	2013

*FRG* Federal Republic of Germany, *GDR* German Democratic Republic, *RKI* Robert Koch Institute

^a^Reintroduction in 1991 after the recommendation was suspended in 1974

In many countries, the introduction of vaccination recommendations is accompanied by the implementation of infectious disease surveillance systems. The primary aim of such surveillance systems is to collect and analyse infectious disease data on an ongoing basis. These data can be used to detect outbreaks and to evaluate the impact of interventions. Surveillance data can be collected through various methods including passive surveillance, active surveillance, and sentinel surveillance (Declich and Carter 1994; MacDonald 2012; Oleckno 2008; Roush 2011):

- Passive surveillance mainly refers to national notification systems based on mandatory case reporting. This means that health care providers (e.g. physicians and hospitals) and laboratories are required by law to routinely report the occurrence of certain infectious diseases to public health officials. Passive surveillance is the most common method for collecting data on vaccine-preventable diseases. It captures the entire population and requires relatively few resources, but completeness of reporting is highly dependent on the compliance of health facilities. Moreover, diagnostic accuracy can differ among health care providers, particularly since case definition criteria of notification systems often include not only laboratory-confirmed cases but also clinically diagnosed cases.
- Active surveillance involves a proactive search for cases by health authorities through contacting health care providers on a regular basis. This data collection procedure can provide a more complete picture of disease frequency, but it is usually more costly than passive surveillance. That is why active surveillance is often limited to outbreaks or other short-term investigations.
- Sentinel surveillance, which may comprise elements of both active and passive surveillance approaches, relies on a limited number of carefully selected reporting sites. Selection criteria may include representativeness, geographic area, and practical considerations related to feasibility and reporting quality (e.g. willingness to participate, well-qualified staff, and adequate technical resources). Sentinel surveillance requires fewer resources than population-based surveillance and can provide high-quality data. The main shortcoming of this approach is that the generalisability of the findings may be limited.

Further data collection procedures include surveillance surveys, epidemic field investigations and the use of secondary data sets (Declich and Carter 1994). In Germany, measles has been a notifiable disease since 2001. Mumps, pertussis, and varicella became notifiable on a national level in March 2013 (Table 1). German sentinel systems were, among others, developed for the surveillance of measles (Siedler and Leitmeyer 2004) and varicella (Siedler and Arndt 2010).

In 2010, the 53 member states of the World Health Organization (WHO) European Region renewed their commitment to the elimination of measles by 2015 (WHO 2010). Nevertheless, the outbreak in Berlin in 2014/2015 (RKI 2015b) clearly demonstrates that Germany has not met the elimination target so far. Therefore, the objective of this study is to systematically review the existing literature on the current epidemiology and economic burden of measles and other childhood diseases (namely mumps, pertussis, and varicella) in Germany.

Epidemiological measures of interest include incidence, frequency of complications and long-term sequelae, mortality, outbreak descriptions, and disability-adjusted life years (DALYs). The DALY is a summary measure of population health that captures the burden of morbidity and mortality in a single metric. DALYs for a particular disease are calculated as the sum of the time lost due to premature death and the time lived with poor health or disability. The concept includes weighting of disease duration with a weight factor that reflects the severity of the health condition (Murray 1994). Since the development of this measure in the early 1990s, the methods used to calculate DALYs have undergone several changes (Devleesschauwer et al. 2014; Murray et al. 2012; Voigt and King 2014). The DALY approach is primarily used by the WHO to quantify the global burden of disease.

Our definition of economic burden covers resource consumption (particularly hospitalisation), illness-related work days lost, and direct and indirect costs. Direct costs primarily capture the cost of medical care (e.g. drug therapy, physician consultations, inpatient treatment), whereas indirect costs mainly refer to productivity losses due to absence from work and premature death.

## Methods

### Literature search

We searched in the literature databases PubMed and EMBASE for relevant papers published between 1 January 2000 and 8 February 2015. Search terms included controlled vocabulary and free-text terms. Details of the search strategy are provided in the supplementary material (Online Resource 1). Two investigators (OD and JW) independently screened search results and assessed the eligibility of potentially relevant studies according to predefined inclusion and exclusion criteria. Discrepancies were solved through discussion involving a third investigator (SW). Reference lists of identified studies were searched manually for further relevant publications. We followed the PRISMA guidelines for conducting and reporting systematic reviews (Moher et al. 2009).

### Inclusion and exclusion criteria

We included English and German language articles reporting data on the epidemiology and economic burden of measles, mumps, pertussis, and varicella in Germany. We did not apply restrictions concerning the type of study and the method of data collection. We wanted to focus as far as possible on the general population and, therefore, excluded studies restricted to health care workers, military personnel and their families. We also excluded pure review articles, comments, letters, editorials, single case-reports, small case series and outbreak reports with fewer than 10 subjects, articles without full-text (e.g. conference abstracts), and surveillance and outbreak reports lacking a separate methods section.

### Data extraction

Data extraction was performed by one investigator (OD) and verified by a second (JW). The following data were abstracted from the included studies: type of study, data sources and methods, population and setting, time frame, outcome measures, and results.

### Surveillance data

In addition to our literature search, we used notification data of the German Protection against Infection Act (Infektionsschutzgesetz) to give an up-to-date overview on the epidemiology of measles, mumps, pertussis, and varicella in Germany. The numbers of notified cases were extracted using a web-based data query tool (SurvStat@RKI 2.0).

## Results

### Literature review

**Fig. 1:**
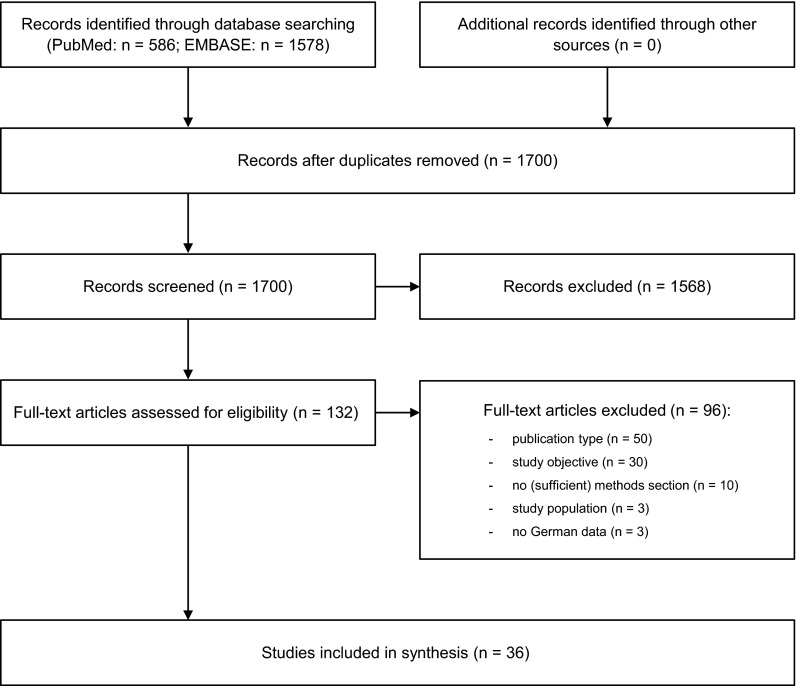
Flowchart of the study identification and selection process

After duplicates were removed, the bibliographic search using PubMed and EMBASE databases yielded 1700 records. After screening of titles and abstracts, 1568 records were excluded and 132 full-text articles were subsequently assessed for eligibility. Ninety-six articles were excluded after full-text assessment. Main reasons for exclusion were study objective (e.g. vaccination coverage or vaccine effectiveness), publication type (e.g. review article, case report, or comment), or lack of a sufficient methods section. Thirty-six studies fulfilled the inclusion criteria and were, therefore, included in this review. Figure 1 shows the process of study identification and selection.

Of the 36 included studies, 18 reported results for measles, two for mumps, six for pertussis, and ten for varicella. Most of the studies were mainly based on data from (mandatory) notification and/or sentinel surveillance systems. Four of these studies used data from a paediatric hospital-based surveillance system, called ESPED (Erhebungseinheit für seltene pädiatrische Erkrankungen in Deutschland; German paediatric surveillance unit). Three studies used claims data of the Associations of Statutory Health Insurance Physicians (ASHIP). Two studies explicitly applied (decision-analytic) modelling approaches. Other methods and data sources used include survey and interview methods, contact tracing activities, and review of medical files or national statistics. The majority of the included studies (*n* = 34) provided data on epidemiological outcomes and/or hospitalisation. Only few studies reported on economic aspects in terms of costs and/or work days lost (*n* = 5). Table 2 summarises the characteristics and results of the included studies. The main results are also described in the following paragraphs.

**Table 2:** Study characteristics and results

Publication	Methods (study type, data source, population, and time frame)	Outcome measures	Results
Measles			
Arenz et al. (2009)	Analysis of surveillance data; paediatric hospital-based surveillance data (ESPED); hospitalised children and adolescents <16 years (detailed questionnaire-based information was obtained for 96 children); 2006	Children hospitalised for measles	115 children (42 % were <2 years)
		Median length of hospital stay	6 days
		Complications	Pneumonia: 54 %; otitis media: 11 %; seizures: 7 %
		Measles-related deaths	2 children died of measles (with encephalitis)
Carabin et al. (2003)	Multi-country cost study; country-specific incidence and cost data; direct costs include physicians’ visits, prescription medication, hospitalisation, and long-term care for sequelae; general population in Germany; 2001	Average annual costs (2001 values) of caring for measles per capita in Germany from a health care provider perspective	Approximately EUR 0.02 per capita
Gillesberg Lassen et al. (2014)	Outbreak report; notification data and clinical data collected through interviews with case-patients; community members, students of an anthroposophic school, and family members and friends of the students in Berlin; April–July 2011	Cases of measles	73 cases (27 % of all case-patients and 38 % of community case-patients were ≥20 years)
		Hospitalisation	15 %
Hegasy et al. (2012)	Outbreak report; notification data and contact tracing activities; non-Roma inhabitants and Roma community members living in Hamburg; December 2008–June 2009	Cases of measles	216 cases (69 % were confirmed by laboratory analyses); a local Roma community comprised more than 50 % of the notified cases
Mankertz et al. (2011)	Study on spread of the D4-Hamburg strain; results of laboratory samples; general population in Germany; 2008–2010	Cases of measles	216 cases in Hamburg; 72 cases in Lower Saxony; 48 cases in Munich; several cases occurred in Roma community members and asylum seekers
		Hospitalisation	40 % of patients (due to pneumonia or otitis media)
Mette et al. (2011)	Claims data analysis and comparison with surveillance data; ASHIP billing data and notification data; 15.4 million people covered by statutory health insurance living in North Rhine-Westphalia; 2006–2007	Confirmed measles diagnoses (ASHIP data)	2534 diagnoses (87 % of billed measles diagnoses occurred in children <10 years)
		Reported cases of measles (notification data)	2014 cases (40 % of measles cases were reported for children <10 years)
		Ratio of confirmed measles diagnoses and reported cases of measles	1.26: 1 (underreporting)
Muscat et al. (2009)	Multi-country analysis of surveillance data; data of national mandatory notification systems; general population in Germany; 2006–2007	Cases of measles	2307 cases in 2006; 571 cases in 2007
		Incidence per 100,000 inhabitants	2.8 per 100,000 in 2006; 0.7 per 100,000 in 2007
		Measles-related deaths	2 fatal cases in children (caused by encephalitis)
Muscat et al. (2014)	Multi-country analysis of surveillance data; data submitted by national surveillance institutions to the WHO Regional Office for Europe; general population in Germany; 2012–2013	Cases of measles	167 cases in 2012; 1773 cases in 2013
		Incidence per million inhabitants per year	2 per million in 2012; 21.4 per million in 2013
Plass et al. (2014)	Burden of disease study; DALY estimates are based on a model of the natural history of disease using notification data; general population in Germany; 2005–2007	Average loss of DALYs per year	740 DALYs (93 % was due to acute symptomatic infections and 7 % was due to long-term sequelae)
		Average loss of DALYs per case	0.26 DALYs
Roggendorf et al. (2012)	Outbreak report; surveillance data of a community health centre; 1st outbreak: children attending a free progressive school in Essen and their contacts; 2nd outbreak: children and adults in a low socio-economic setting and with migration background in Essen; March–July 2010	Cases of measles	1st outbreak: 75 cases; 2nd outbreak: 11 cases
		Hospitalisation	15 %
Siedler et al. (2006)	Outbreak report; surveillance data of the mandatory reporting system and data collected through interviews of the local health authorities with physicians and family members; general population in Hesse and Bavaria; January–May 2005 (Hesse) and March–July 2005 (Bavaria)	Cases of measles (without sporadic cases)	Hesse: 223 cases; Bavaria: 279 cases; 74 % in school aged children
		Incidence per 100,000 inhabitants	Hesse: 14 per 100,000; Bavaria: 12 per 100,000
		Hospitalisation in patients ≥20 years	Outbreak in Hesse: 34 %
		Measles-related deaths	Outbreak in Hesse: 1 case
Siedler et al. (2013a)	Analysis of surveillance data; sentinel data collected by 1488 paediatric and primary care practices; patients of practices participating in the sentinel system; 2001–2010	Cases of measles	3100 cases (2495 cases in children <10 years)
		Complications	15 % (mostly otitis media and pneumonia)
Takla et al. (2014)	Claims data analysis and comparison with surveillance data; ASHIP billing data and mandatory notification data; 68 % (2007) and 79 % (2008–2011) of the total population living in Germany (ASHIP data); 2007–2011	Cases of measles (notification data)	4440 cases
		Annual incidence per million population (notification data)	Total: 10.8 per million (range 6.9–19.6 per year); northern Germany: 8.7; western Germany: 7.2; eastern Germany: 5.5; southern Germany: 20.3
		Annual incidence per million residents with statutory health insurance (ASHIP data)	27.5 per million; incidence based on ASHIP data was up to 4.8-fold higher than incidence based on notified cases
Tischer et al. (2001)	Analysis of surveillance data; sentinel data and notification data; general population in Germany; October 1999–March 2001	Cases of measles (sentinel data)	1291 cases
		Complications (sentinel data)	24 %
		Hospitalisation (sentinel data)	2.2 %
		Incidence per 100,000 inhabitants	38.9 per 100,000 (sentinel data); <0.5–5.7 per 100,000 (notification data of the first quarter of 2001)
Tischer et al. (2002)	Analysis of surveillance data; sentinel data collected by 1271 paediatric and primary care practices; general population in Germany; October 1999–September 2001	Cases of measles	1720 cases
		Incidence per 100,000 inhabitants	20 per 100,000 (range <1–56 per 100,000, depending on the federal state)
		Complications	16 % (mostly otitis media and pneumonia)
		Hospitalisation	2.4 %
Wadl et al. (2011)	Outbreak report; surveillance data and data collected through questionnaires; general population in four Bavarian counties (including attendees of an anthroposophic school in Austria); March–July 2008	Cases of measles	217 cases
		Incidence per 100,000 population	32 per 100,000
		Hospitalisation	11 %
		Complications	18 %
Wichmann et al. (2007)	Retrospective cohort study on the initial phase of an outbreak; data collected through questionnaires; 1098 students aged 10–21 years of a public day school in Duisburg; January–May 2006	Cases of measles	53 cases
		Hospitalisation	4 %
		Complications	Otitis media: 4 cases; pneumonia: 1 case; encephalitis: 1 case
Wichmann et al. (2009)	Study on outbreak-related morbidity and costs; surveillance data and data collected through questionnaires/interviews (face-to-face or by telephone); health care provider costs (including physician consultations, laboratory tests, antibiotic treatment, and hospitalisation) are calculated using DRGs, the outpatient fee schedule and medication prices; general population in Duisburg; 2006	Cases of measles	614 cases in Duisburg
		Hospitalisation	15 %
		Antibiotic treatment	32 %
		Complications	Otitis media: 19 %; pneumonia: 7 %; encephalitis: 0.6 %
		Measles-related deaths	2 fatal cases in children (caused by encephalitis)
		School days missed	2854 days
		Work days lost	301 days
		Average costs (2006 values)	EUR 373 per measles patient; EUR 1877 per hospitalised patient
Mumps			
Otto et al. (2010)	Outbreak report; laboratory samples and clinical data; adolescents and young adults in Bavaria; July–October 2010	Laboratory-confirmed infections	115 laboratory-confirmed mumps infections (median age: 24.5 years, predominantly male patients)
		Complications	1 case of meningitis and 21 cases of orchitis
Takla et al. (2013)	Claims data analysis and comparison with surveillance data; ASHIP billing data and notification data; statutory health-insured population and general population in the eastern and western federal states of Germany; 2007–2011	Countrywide mean annual incidence per 100,000 people covered by statutory health insurance	10.3 per 100,000 (range 9.3–11.8); incidence was significantly higher in western than in eastern federal states; comparison of notification data with ASHIP data indicated severe underreporting of incidence estimates based on notification data
		Complications	Orchitis: 6.2 % of male cases; meningitis: 0.4 %; pancreatitis: 0.3 %; encephalitis: 0.2 %; proportion of complications in cases ≥15 years was significantly higher than in cases <15 years
Pertussis			
Hellenbrand et al. (2009)	Analysis of surveillance data; surveillance data (notification and sentinel data) and hospital discharge statistics; general population in Germany; 2000–2007	Incidence per 100,000 inhabitants in eastern federal states (notification data)	20.5 per 100,000 in 2000; 39.3 per 100,000 in 2007
		Incidence in adults per 100,000 inhabitants	160–169 per 100,000 in 2002–2004
		Hospitalisation in eastern federal states (surveillance data)	1.9–4.9 % in 2002–2007 (depending on the federal state)
		Hospitalisation (cases per 100,000 population, hospital discharge statistics)	1.7 per 100,000 in eastern federal states in 2007; 1.5 per 100,000 in western federal states in 2005; most cases occurred in children <1 year
Juretzko et al. (2001)	Analysis of surveillance data; paediatric hospital-based surveillance data (ESPED) and clinical data collected through questionnaires; children <16 years; 1997–1998	Standardised incidence of pertussis requiring hospitalisation per 100,000 person-years	2.70 per 100,000; 2.36 per 100,000 in western federal states; 4.50 per 100,000 in eastern federal states
		Mean length of hospital stay	14.9 days
		Complications in hospitalised children	All: 44 % (60 % occurred in children <6 months); pneumonia: 28.1 %; apnea: 20.6 %; seizures: 2.5 %; encephalopathy: 2.1 %
		Pertussis-related deaths in hospitalised children	0.3 %
Liese et al. (2003)	Prospective long-term surveillance study; follow-up data of a population-based case–control efficacy study; 11,087 children (3–8 years) of the original study population and all other children of the same age group presenting in the participating paediatric practices; 8.3 % were not vaccinated against pertussis; May 1997–March 1999	Cases of pertussis	180 cases; 64 % were caused by *B pertussis* infections and 36 % were caused by *B parapertussis* infections
		Incidence per 1000 person-years	*B pertussis* infections: 4.8 per 1000; *B parapertussis* infections: 2.8 per 1000
Riffelmann et al. (2006)	Prospective incidence and cost study; laboratory samples, clinical and resource consumption data collected through questionnaires; direct costs include physician consultations, laboratory tests, and medication; indirect costs are based on the number of work days lost and a cost of EUR 114.30 per day; 971 primary care patients having cough for ≥7 days in two German cities (Krefeld and Rostock); economic analysis is based on 45 cases of pertussis; 2001–2004	Proportion of patients with pertussis	10 %
		Incidence per 100,000 inhabitants	165 per 100,000
		Pertussis patients with antibiotic prescription	53 %
		Average direct costs (2004 values) per case	EUR 120
		Average indirect costs (2004 values) per case in employed patients	EUR 2443
Sin et al. (2009)	Outbreak report; active case finding by the local health authorities and a retrospective cohort study performed in 4 affected schools (questionnaires); mostly children and adolescents attending schools in Ludwigslust district, Mecklenburg-Western Pomerania; 2005–2006	Cases of pertussis	104 cases
		Attack rate	1.9–32.0 % (depending on the time since last vaccine dose); results suggest that vaccine-induced immunity begins to wane approximately 5 years after completion of the primary series
Stojanov et al. (2000)	Prospective surveillance study; data of a case–control efficacy study; 11,016 children <2 years presenting with cough ≤7 days at 63 paediatric practices in Germany; March 1993–May 1995	Proportion of patients with pertussis	6.6 %
		Hospitalisation	4.5 %
		Complications in hospitalised pertussis patients	All: 48 %; bradycardia: 21 %; apnea: 12 %; conjunctivitis: 12 %; pneumonia: 6 %; otitis media: 6 %
		Mean length of hospital stay	8 days
Varicella			
Banz et al. (2004)	Cost study; cost estimates are based on a decision-analytic model using survey data (Wagenpfeil et al. 2004); direct costs include physician consultations, medication, and hospitalisation; transfer payments are based on parental work days lost; indirect costs are based on the number of work days lost and a cost of EUR 150 per day; general population in Germany; 1999 prices (pre-vaccination era)	Annual third-party payer costs (1999 values of direct costs and transfer payments to parents caring for their sick children)	EUR 78 million (direct medical costs: 43 %)
		Annual societal costs (1999 values of direct and indirect costs)	EUR 187.5 million (direct medical costs: 18 %)
Grote et al. (2007)	Analysis of surveillance data; paediatric hospital-based surveillance data (ESPED); paediatric population up to the age of 17 years; 2003–2004 (pre-vaccination era)	Cases of varicella-associated deaths	10 cases (none was vaccinated against varicella)
		Annual mortality rate (cases per million children)	0.4 per million
Liese et al. (2008)	Analysis of surveillance data; nationwide paediatric hospital-based surveillance data (ESPED), practice sentinel network and hospital diagnosis data in one federal state (North Rhine-Westphalia); children ≤16 years; 2003–2004 (pre-vaccination era)	Hospitalised varicella cases (ESPED data)	918 cases (with a median age of 3.3 years)
		Annual incidence of varicella-related hospitalisations (cases per 100,000 children)	3.25 per 100,000 (ESPED data); 14.1 per 100,000 (capture–recapture methodology for two sources); 19.7 per 100,000 (hospital diagnosis data)
		Median length of hospital stay	5 days
		Complications in hospitalised cases	All: 79.6 %; neurological: 25.4 %; skin: 23.2 %; gastrointestinal tract: 15 %; lower respiratory tract: 11.8 %; severe systemic bacterial infections: 4.4 %
		Cases of varicella-associated deaths	10 cases
Siedler and Arndt (2010)	Analysis of surveillance data; sentinel data collected by paediatricians and general practitioners; general population in Germany; April 2005–March 2009 (vaccination era)	Cases of varicella and trend analysis	83,181 cases; sentinel data showed a reduction of 55 % of varicella cases in all ages over time
Siedler et al. (2013b)	Analysis of surveillance data; sentinel data, notification data, and hospital diagnosis statistics; general population in Germany; 2005–2012 (vaccination era)	Trend analysis of varicella-related morbidity	Significant decline of varicella incidence, complications, and hospitalisations over time
Siedler and Dettmann (2014)	Analysis of hospitalisation data; national hospital discharge statistics; general population in Germany; 1995–2012 (pre-vaccination era and vaccination era)	Trend analysis of varicella hospitalisation incidence	No particular trend until 2003, hospitalisation incidence peaked in 2004 (time of vaccine recommendation), and decreased thereafter; hospitalisation incidence per 100,000 was significantly lower in the vaccination period (1.86) than in the pre-vaccination period (3.30)
Spackova et al. (2010)	Analysis of surveillance data; sentinel data collected by paediatricians and general practitioners; general population in Germany; April 2005–March 2009 (vaccination era)	Cases of varicella and trend analysis	83,075 cases; the total number of varicella cases decreased over time with increasing vaccine uptake
		Complications	All: 0,34 %; bacterial superinfection: 0.13 %; otitis media: 0.06 %; neurological: 0.03 %
		Hospitalisation in varicella patients with complications	25 %
Streng et al. (2013)	Analysis of surveillance and survey data; data of the Bavarian Varicella Surveillance Project (BaVariPro) based on parent surveys (vaccination coverage), paediatric practice surveillance, and paediatric hospital database queries; children <17 years in Munich; October 2006–September 2011 (vaccination era)	Cases of varicella and trend analysis	16,054 cases; the mean number of cases decreased by 67 % during the five seasons
		Incidence per 1000 children (based on the number of reported cases)	26 per 1000 in the first season and 6 per 1000 in the fifth season
		Complications in practice patients	0.8 % (mostly skin complications and otitis media)
		Complications in hospitalised patients	Central nervous system: 22.5 %; skin: 15.7 %; lower respiratory tract: 9 %
		Median length of hospital stay	3 days
		Hospitalisation incidence (cases per 100,000 children) and trend analysis	8.2 per 100,000; hospitalisation incidence decreased by 43 % between 2005 and 2009
Wagenpfeil et al. (2004)	Retrospective epidemiological and resource consumption study; clinical data were obtained from medical files through telephone interviews with physicians; 1334 unvaccinated varicella patients of a representative and German-wide sample of paediatric and primary care practices; 1999 (pre-vaccination era)	Complications	All: 5.7 %; bacterial superinfection: 2.5 % (≤12 years), 1.9 % (>12 years); neurological: 0.2 % (≤12 years); pneumonia or bronchitis: 1.9 % (≤12 years), 1.9 % (>12 years); otitis media: 1.1 % (≤12 years)
		Hospitalisation period per diagnosed varicella case	0.1 inpatient days
		Work days lost per diagnosed varicella case	1.3 days
Ziebold et al. (2001)	Analysis of surveillance data; paediatric hospital-based surveillance data (ESPED) and clinical data collected through questionnaires; hospitalised children ≤16 years; 1997 (pre-vaccination era)	Hospitalised varicella cases	119
		Complications in hospitalised varicella cases	Neurological: 62.3 %; infectious: 35.2 %

*ASHIP* Associations of Statutory Health Insurance Physicians, *BaVariPro* Bavarian Varicella Surveillance Project, *DALY* disability-adjusted life year, *DRG* diagnosis-related group, *ESPED* Erhebungseinheit für seltene pädiatrische Erkrankungen in Deutschland (German paediatric surveillance unit), *WHO* World Health Organization

#### Measles

Incidence estimates based on analyses of surveillance data ranged widely across studies and years (from <0.1 to 38.9 per 100,000 inhabitants per year). Outbreak-related incidence estimates ranged between 12 and 32 per 100,000 inhabitants. One study examining the epidemiology of measles from 2007 to 2011 (Takla et al. 2014) showed large geographical differences in incidence of measles with the highest incidence in southern Germany. Another study presenting results for 1999 to 2001 (Tischer et al. 2002) found that most cases of measles occurred in southern Germany and in North Rhine-Westphalia. Two studies (Mette et al. 2011; Takla et al. 2014) compared results of the analyses of surveillance data and ASHIP claims data and found that incidence estimates based on ASHIP data were higher than estimates based on notification data. Some studies only reported absolute numbers of cases instead of presenting data as incidence rates (see Table 2 for details). The proportion of patients developing any complications varied from 15 to 24 %. The most frequent complications of measles were otitis media and pneumonia. In hospitalised children, pneumonia was diagnosed in more than 50 % of the patients (Arenz et al. 2009). The proportion of measles-associated hospitalisations ranged from 2.2 to 40 % in studies examining all ages. One study found a proportion of hospitalisation of 34 % in patients ≤20 years (Siedler et al. 2006). Median length of hospital stay in children was 6 days (Arenz et al. 2009). Four studies provided information on measles-related deaths, which occurred rarely and only in children (see Table 2 for details). In a German substudy of the Burden of Communicable Diseases in Europe (BCoDE) project, the average loss of DALYs per case of measles was estimated to be 0.26 resulting in an average DALY loss per year of 740 (Plass et al. 2014). The average costs per measles patient and per hospitalisation were EUR 373 and 1877, respectively (Wichmann et al. 2009).

#### Mumps

Mean annual incidence of mumps based on claims data was estimated to be 10.3 per 100,000 people covered by statutory health insurance. Incidence was significantly higher in western federal states than in eastern federal states. A comparison between claims data and notification data indicated severe underreporting of mumps incidence in the notification surveillance system (see Table 2 for details). The main complication was orchitis affecting 6.2 % of male cases. The proportion of complications in individuals ≥15 years was higher than in younger patients (Takla et al. 2013). Information on the economic burden of mumps was not available.

#### Pertussis

In children, incidence of infections with *Bordetella pertussis* and *Bordetella parapertussis* was 4.8 and 2.8 per 1000 person-years, respectively. More than 60 % of all pertussis cases in children from 3 to 8 years were caused by *Bordetella pertussis* (Liese et al. 2003). Incidence of pertussis requiring hospitalisation was 2.7 per 100,000 person-years in children (Juretzko et al. 2001). In adults, incidence of pertussis ranged from 160 to 169 per 100,000 inhabitants (Hellenbrand et al. 2009). The diagnosis of pertussis could be verified in 10 % of the primary care patients having cough for ≥7 days (Riffelmann et al. 2006). In children <2 years presenting with cough for ≥7 days, pertussis was diagnosed in 6.6 % of the cases (Stojanov et al. 2000). Pertussis-associated hospitalisation rate was 1.5 and 1.7 per 100,000 population in the western and eastern federal states, respectively (Hellenbrand et al. 2009). Most of the hospitalisations occurred in children <1 year (Juretzko et al. 2001). More than 40 % of hospitalised children suffered from complications, and the mean length of hospitalisation in children varied between 8 and 14.9 days (Juretzko et al. 2001; Stojanov et al. 2000). Direct costs per case of pertussis were EUR 120 in primary care patients, and indirect costs per case were EUR 2443 in employed patients (Riffelmann et al. 2006). None of the reviewed studies focused on older adults (>60 years).

#### Varicella

Studies analysing surveillance data from the vaccination era showed a decline of varicella incidence and hospitalisations over time and with increasing vaccine uptake (Siedler and Arndt 2010; Siedler et al. 2013b; Siedler and Dettmann 2014; Spackova et al. 2010; Streng et al. 2013). The proportion of patients with complications was higher in the pre-vaccination era (5.7 %) (Wagenpfeil et al. 2004) than in the vaccination era (0.34–0.8 %) (Spackova et al. 2010; Streng et al. 2013). Annual incidence of varicella-related hospitalisations varied from 3.25 to 19.7 per 100,000 children depending on the data source used before routine childhood vaccination against varicella was implemented (Liese et al. 2008). About 80 % of these (hospitalised) children suffered from varicella-related complications, and most frequent complications were neurological and infectious complications (Liese et al. 2008; Ziebold et al. 2001). In the pre-vaccination era, annual varicella-associated mortality in children was 0.4 per million (Grote et al. 2007), and societal costs of varicella were estimated at EUR 187.5 million per year (Banz et al. 2004). However, direct medical costs accounted for only 18 % of these costs. Work days lost per diagnosed varicella case were 1.3 days (Wagenpfeil et al. 2004).

### Analysis of surveillance data

**Fig. 2:**
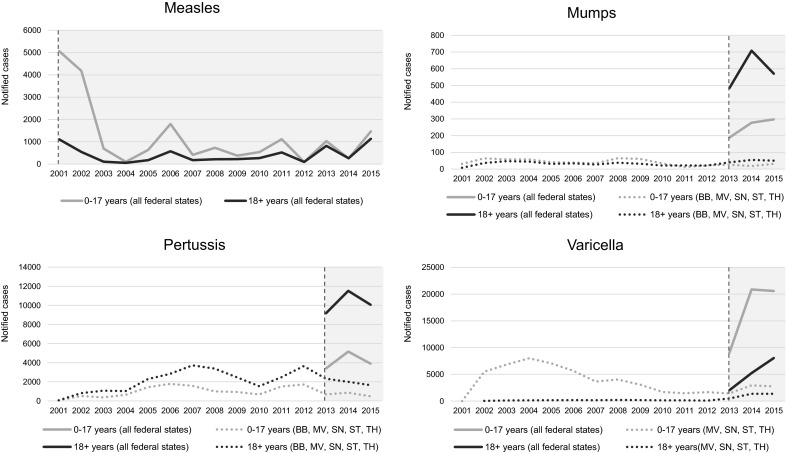
Numbers of notified cases of measles, mumps, pertussis, and varicella in Germany (2001–2015) based on SurvStat@RKI 2.0. *Vertical dashed lines* indicate the date of nationwide implementation of mandatory notification. *BB* Brandenburg, *MV* Mecklenburg-West Pomerania, *SN* Saxony, *ST* Saxony-Anhalt, *TH* Thuringia

The numbers of reported cases of measles, mumps, pertussis, and varicella over time are shown in Fig. 2. Nationwide mandatory notification of measles was introduced in 2001. Since then, the number of reported cases of measles varied from year to year. Mumps, pertussis, and varicella became officially notifiable diseases in Germany in 2013. Hence, the numbers of notified cases of these diseases strongly increased from that point of time. In 2014, about 70 % of all reported mumps and pertussis cases occurred in adults, while most of the notified varicella cases occurred in children. For the period before 2013, notification data on mumps, pertussis, and varicella were available only for federal states in the eastern part of Germany (see dotted lines in Fig. 2).

## Discussion

Our review examined available information on the epidemiology and economic burden of measles, mumps, pertussis, and varicella in Germany. In general, results differed widely by reporting year, population, and data source used. More specific key findings are discussed below.

Results of the studies investigating the measles epidemiology in Germany and latest reports on the ongoing outbreak in Berlin (RKI 2015b) clearly showed that Germany has failed in achieving the aim of eliminating measles until 2015. Some of the reviewed studies found huge regional difference in measles incidence. Most cases occurred in the context of regionally limited outbreaks, and several outbreaks were linked to transmission from Roma community members, asylum seekers, or anthroposophic communities/schools. These findings are in line with a recently published analysis of the measles epidemiology in 2014/2015 by the RKI (2015b) and indicate the importance of conducting more in-depth analyses at a regional level, increasing public awareness on the benefits of immunisation, and strengthening efforts to identify and close the existing vaccination gaps. The relevance of targeting vulnerable groups such as Rome communities, anthroposophic groups, and immigrants has already been emphasised by an overview of affected groups in Europe (Muscat 2011). Comparative analyses of different data sources (notification data vs. claims data) revealed a potential underestimation of measles incidence estimates when using notification data. Hence, claims data analyses should be used more regularly to complement analyses based on surveillance systems and to provide a more comprehensive picture of the epidemiology of notifiable diseases in Germany.

Compared to measles, only very few studies have dealt with the epidemiology of mumps in Germany. However, some of the findings concerning measles also hold true for mumps such as the underreporting of incidence estimates based on surveillance data. Another similarity was found between mumps and pertussis: in 2014, about 70 % of all notified cases occurred in adults. Studies from other countries have also reported an increasing incidence of pertussis in adolescents and adults (Rothstein and Edwards 2005; McGuiness et al. 2013). Moreover, there is evidence that adults are one of the major sources of pertussis in infants (Orenstein 1999; Bisgard et al. 2004) and play a crucial role in transmitting pertussis to household members (Baptista et al. 2009). The reason for the age shift of pertussis is supposed to be the combination of less boosting by natural infections and waning of vaccine-induced immunity (Nitsch-Osuch et al. 2013). One of the reviewed studies showed that pertussis was a common cause of persistent cough in adults, which is in line with published data from other countries (Rothstein and Edwards 2005). In general, it is assumed that standard surveillance systems greatly underestimate the level of pertussis (Crowcroft and Pebody 2006).

Several of the included studies on varicella reported on the situation in the pre-vaccination era. Studies that are more current showed that varicella incidence and hospitalisation have decreased after the implementation of routine childhood vaccination. Similar effects have been observed after the introduction of the varicella vaccination programme in the United States (Marin et al. 2008; Baxter et al. 2014). Furthermore, the overall decline in varicella incidence and hospitalisation in the United States was not associated with a shift to older age groups (Baxter et al. 2014).

Our review revealed that information on direct and indirect costs of childhood diseases in Germany is scarce. Furthermore, not all studies that collected cost data included all relevant cost components. For example, in the study by Riffelmann et al. (2006), direct costs were calculated without considering hospitalisation cost. In contrast, the study by Banz et al. (2004) considered an additional cost category when adopting a third-party payer perspective, namely transfer payments to parents that stay at home to care for their sick children. Inclusion of this cost category can substantially increase costs from the health care payer perspective, particularly when assuming that all sick children cause parental absence from work. For instance, in the study by Banz et al. (2004), the mean number of parental work days lost per sick child ranged from 0.6 to 4 depending on the course of disease, and as a consequence, the reimbursed costs of parental work days lost accounted for 57 % of the total third-party payer costs.

The use of different outcome measures also hampers the comparison of economic results across studies. The study by Carabin et al. (2003), which estimated the costs of measles for 11 countries, used per capita costs (approximately EUR 0.02) as economic outcome measure, whereas the study by Wichmann et al. (2009) presented results in terms of costs per measles patient (EUR 373) and costs per hospitalised patient (EUR 1877).

In summary, since only few studies have provided cost estimates so far, future research should concentrate on quantifying the economic burden of disease. A more intensified use of claims data analyses might contribute to this aim. Also, administrative data from health insurance funds might provide a good foundation to supplement surveillance data (Jones et al. 2013) and to extend existing methods of measuring underreporting of notified cases of infectious diseases (Gibbons et al. 2014). However, as current case definitions include not only laboratory-confirmed cases but also clinically diagnosed cases (RKI 2015a), notification data might also be subject to overestimation. Certainly, the same is true for health insurance claims data, since the validity of the recorded diagnoses is largely unknown.

### Limitations

There are several limitations of our systematic review. First, since the scope of our review was limited to studies presenting results for Germany, transferability of results to other countries is limited, too. Second, due to the high heterogeneity of the included studies, an assessment of methodological quality was not performed. Third, many of the included studies were based on surveillance data that might be subject to underreporting. Fourth, case definitions varied among studies, which might partially explain differences in results.

### Conclusions

This review aimed to provide an overview of the epidemiology and economic burden of measles, mumps, pertussis, and varicella in Germany. Most of the reviewed studies presented epidemiological outcomes. Studies providing information on economic aspects except hospitalisation were scarce.

Despite the existing immunisation recommendations, results suggest that there is still considerable morbidity due to childhood diseases in Germany. However, not only children are affected. For instance, a high proportion of all pertussis cases occurs in adults. Furthermore, several studies revealed regional differences in incidence of some of the target diseases. These findings underline the need for improved vaccination and communication strategies targeting all susceptible age and risk groups on a national and local level.

## Electronic supplementary material

Below is the link to the electronic supplementary material.

Supplementary material 1 (PDF 187 kb)
