# Supplementary material for: Epidemiology and economic burden of measles, mumps, pertussis, and varicella in Germany: a systematic review
Source: Int J Public Health. 2016 Aug 4;61(7):847–60. doi: 10.1007/s00038-016-0842-8 (PMC5002040; doi:10.1007/s00038-016-0842-8)
Supplement: Supplementary file 1 — Supplementary material 1 (PDF 187 kb) [file 38_2016_842_MOESM1_ESM.pdf]

International Journal of Public Health

Supplementary material to:

Epidemiology and economic burden of measles, mumps, pertussis, and varicella in Germany: a systematic review

Oliver Damm, Julian Witte, Stefanie Wetzka, Christine Prosser, Sebastian Braun, Robert Welte, Wolfgang Greiner

Author for correspondence: Oliver Damm, Department of Health Economics and Health Care Management, School of Public Health, Bielefeld University, Bielefeld, Germany

Email: oliver.damm@uni-bielefeld.de

Search strategy

Database	Search terms		
	Disease[a]	Epidemiology and economic burden[a]	Germany
PubMed	"measles"[MeSH] OR "mumps"[MeSH] OR "whooping cough"[MeSH] OR "chickenpox"[MeSH] OR "measles" OR "morbilli" OR "rubeola" OR "mumps" OR "epidemic parotitis" OR "pertussis" OR "whooping cough" OR "varicella" OR "chickenpox" OR "chicken pox"	"epidemiology"[MeSH] OR "epidemiologic studies"[MeSH] OR "epidemiology" OR "incidence" OR "prevalence" OR "burden" OR "seroprevalence" OR "rate" OR "rates" OR "epidemiologic" OR "epidemiological" OR "sequelae" OR "complication" OR "complications" OR "outbreak" OR "outbreaks" OR "population" OR "survey" OR "cohort" OR "observational" OR "claims data" OR "costs and cost analysis"[MeSH] OR "cost" OR "costs" OR "economic" OR "hospitalization" OR "hospitalizations" OR "hospitalisation" OR "hospitalisations" OR "consultation" OR "consultations" OR "work loss" OR "productivity losses" OR "productivity loss" OR "production losses" OR "production loss" OR "absenteeism"	"Germany" OR "German" OR "Deutschland" OR "deutsche" OR "deutsches" OR "deutscher" OR "deutschen" OR "Berlin" OR "Hamburg" OR "Munich" OR "Munchen" OR "Cologne" OR "Koln" OR "Frankfurt" OR "Stuttgart" OR "Dusseldorf"
EMBASE	"pertussis"/exp OR "measles"/exp OR "mumps"/exp OR "chickenpox"/exp OR "measles" OR "morbilli" OR "rubeola" OR "mumps" OR "epidemic parotitis" OR "pertussis" OR "whooping cough" OR "varicella" OR "chickenpox" OR "chicken pox"	"epidemiology"/exp OR "epidemiological data"/exp OR "complication"/exp OR "epidemic"/exp OR "population and population related phenomena"/exp OR "health care survey"/exp OR "observational study"/exp OR "cohort analysis"/exp OR "epidemiology" OR "incidence" OR "prevalence" OR "burden" OR "seroprevalence" OR "rate" OR "rates" OR "epidemiologic" OR "epidemiological" OR "sequel" OR "complication" OR "complications" OR "outbreak" OR "outbreaks" OR "population" OR "survey" OR "cohort" OR "observational" OR "claims data" OR "economic aspect"/exp OR "cost" OR "costs" OR "economic" OR "hospitalization" OR "hospitalizations" OR „hospitalisation" OR "hospitalisations" OR "consultation" OR "consultations" OR "work loss" OR "productivity losses" OR "productivity loss" OR "production losses" OR "production loss" OR "absenteeism"	"Germany" OR "German" OR "Deutschland" OR "deutsche" OR "deutsches" OR "deutscher" OR "deutschen" OR "Berlin" OR "Hamburg" OR "Munich" OR "Munchen" OR "Cologne" OR "Koln" OR "Frankfurt" OR "Stuttgart" OR "Dusseldorf"

[a] Free-text terms were restricted to title and abstract

'"
